# A high-performance electrochemical aptasensor based on graphene-decorated rhodium nanoparticles to detect HER2-ECD oncomarker in liquid biopsy

**DOI:** 10.1038/s41598-022-07230-3

**Published:** 2022-02-28

**Authors:** Mahdi Sadeghi, Soheila Kashanian, Seyed Morteza Naghib, Elham Arkan

**Affiliations:** 1 Nanobiotechnology Department, Faculty of Innovative Science and Technology, Razi University, Kermanshah, Iran; 2 Faculty of Chemistry, Sensor and Biosensor Research Center (SBRC), Razi University, Kermanshah, Iran; 3 Nano Drug Delivery Research Center, Health Technology Institute, Kermanshah University of Medical Science, 6734667149 Kermanshah, Iran; 4 Biomaterials and Tissue Engineering Research Group, Interdisciplinary Technologies Department, Breast Cancer Research Center (BCRC), Motamed Cancer Institute, ACECR, Tehran, Iran; 5 Nanotechnology Department, School of Advanced Technologies, Iran University of Science and Technology (IUST), 1684613114 Tehran, Iran

**Keywords:** Biochemistry, Biological techniques, Biotechnology, Cancer, Biomarkers, Medical research, Chemistry, Engineering, Materials science, Nanoscience and technology

## Abstract

Evaluation of extracellular domain of human epidermal growth factor receptor-2 (HER2-ECD) oncomarker status is an impressive factor in screening, diagnosing and monitoring early-stage breast cancer (BC). Electrochemical aptamer-based nanobiosensor with high sensitivity and selectivity for quantitative and qualitative measurement of HER2-ECD oncomarker was developed. In this study, the nanocomposite made by distinct materials included reduced graphene oxide nano-sheets (rGONs) and rhodium nanoparticles (Rh-NPs) on the graphite electrode (GE) surface. This structure resulted in amplified electrochemical activity, high surface area, stability, and bio-compatibility. Each of the steps of preparing nanomaterials and setting up biosensor were carefully examined by analytical and electrochemical techniques. Various modified electrodes were constructed and analyzed in terms of electrochemical performance, morphology, size, and shape of nanomaterials. The GE-based aptasensor had a noteworthy and conducive results against HER2-ECD with a wide dynamic range of 10.0–500.0 ng/mL, a low limit of detection (LOD) of 0.667 ng/mL (significantly less than the clinical cut-off), and a low limit of quantification (LOQ) of 2.01 ng/mL. The benefits provided by this aptasensor such as broad dynamic range, high sensitivity, selectivity, stability, reproducibility, and low cost suggest tremendous potential for non-invasive detection and monitoring of the HER2-ECD levels of BC care and clinical diagnosis.

## Introduction

Nowadays, the incidence of cancer diseases is a major threat to human health^1^. According to statistics reported in 2021, BC with a population of 2.26 million people has the highest number of cancer patients worldwide^2,3^. BC is a complex disease that causes the severe alterations in the levels of genes, proteins, and metabolites^4^. It is impossible to provide adequate and timely treatmentwithout a thorough diagnosis. Sensitive, selective, and rapid diagnostic testing methods not only pave the way for a more effective treatment, but also have a great impact on preventing a variety of cancers, especially BC^5^. Therefore, Medical screening and early detection of BC through quantitative oncomarker measurements are essential to timely follow-up, enhance survival rate, and reduce mortality^6^. Definitive procedures for diagnosing primary stages of BC include mastography, biopsy, ultrasound imaging, and magnetic resonance imaging (MRI). Furthermore, techniques based on gene expression and quantitation such as immunohistochemistry (IHC), radioimmunoassay (RIA), and enzyme immunoassay (EIA) are used to diagnose BC in women^7–9^. However, most of these techniques face several limitations including high complexity, being too expensive, and a significant reduction in sensitivity and specificity^10–12^. For these reasons, researchers have prioritized the development of non-invasive, inexpensive, sensitive, and user-friendly diagnostic technologies to monitor BC at specific times for effective treatment^13^. As a result, novel analytical techniquessuch as point-of-care testing (POCT) for this disease in its early stages provide faster results at the point of care delivery in resource-constrained settingenabling timely and proper treatment.

Electrochemical biosensors have been utilized as a promising diagnostic tool to qualitatively and quantitatively determination of oncomarkers due to their fast reaction, ease of use, low cost, and low detection limit. In fabricating new electrochemical biosensors, it is crucial to select a flawless electrode matrix to optimize the electrochemical response^14–17^. Identifying the oncomarkers used for early detection, recurrence, and monitoring of BC metastasis are necessary. HER2-ECD is one of these oncomarkers in the process of BC diagnosis and treatment through HER2-targeted therapy. The primary mechanism of HER2-ECD receptor overexpression is amplification of its oncogene found on chromosome 17q12. The presence of this oncomarker is essential for the growth and advancement of certain aggressive malignancies such as lung, ovarian, breast, gastric, and oral cancers^18^. HER2-ECD concentration in blood of healthy people and BC patients is in the range of 4.0 to 14 ng/mL and 15 to 75 ng/mL, respectively. which can be used for diagnosis and active surveillance of patients at risk or in treatment^19^. HER2-ECD with higher-than-normal levels are called HER2-positive or HER2^+^. People who are identified as HER2^+^ in BC diagnostic processes, enter the treatment protocol with Herceptin. Herceptin is a monoclonal antibody (Ab) specifically designed and approved to target HER2 receptors on the surface of BC cells and block them from receiving growth signals^20^.

Hybridization of carbon nanomaterials with noble metal nanoparticles providesadvanced biosensors that have preferable optical, mechanical, chemical, and electrical properties^21^. rGONs possesses a high specific surface area, high electrical conductivity (as compared with GONs), excellent biocompatibility, and abundant chemically active sites for chemical functionalization and catalysis. Due to their superior physical and chemical features, rGONs have been extensively used in flexible electronics, batteries, supercapacitors, sensors, and so on. However, rGONs have a propensity to agglomerate or restack through van der Waals forces and pi-pi stacking interactions. Stabilizers or chemical modifiers are usually exploited to enhance the dispersibility of rGONs, but these methods often diminish their analytical performance. Recently, various methods have been developed to prevail over rGONs agglomeration. One of the new strategies involves the assembly of metal nanoparticles (NPs) onto graphene to form hybrid nanocomposites^22,23^.

Rh-NPs have emerged as a desirable and promising strategy to decorate rGONs due to their advantageous properties including a large specific surface area, excellent electrical conductivity, good bio-compatibility, high biomolecular adsorption, and chemical stability^24^. these nanoparticles play a critical role as self-assembled, conductive, and stable monolayers on the surface of nanocomposite.

Various nanomaterials have been investigated to design and fabricate electrochemical biosensors to detect cancerous oncomarkers including multi-branched gold nano-shells and octreotide functionalized Pt nano-flakes modified GCEs for detection of Somatostatin receptors^25^ and nanocomposites of carbon nanotube and polyaniline prepared on the carbon electrode for detecting vascular endothelial growth factor (VEGF)^26^. Biosensors constructed with disposable electrodes such as graphitic electrodes offer low detection limit, easier assembly of metallic NPs, good reproducibility, low contamination and excellent bio-compatibility with antibodies and aptamers^27,28^. Some types of GEs used to diagnostic biosensors are: GE modified by gold NPs for detection of SARS-CoV-2^29^, GE modified with iron oxide/polypyrrole/palladium nanocomposite for detection of methotrexate and folic acid^30^, GE immobilized by antibodies for detection of P53^31^, GE modified by fullerene (C_60_) for detection of Tumorigenicity 2^32^, and GE modified by Fe3O4@ZIF-8 NPs for detection of sumatriptan^33^.

Aptamers possess an extensive variety of medical applications in different fields includingtherapeutic solutions, diagnostic services, and biosensors^34^. Their recognition properties are determined not only by their specific sequences, but also by their ability to fold into distinct shapes with specified functions. These outstanding features make the aptamers as good candidates for the bio-recognition agents of electrochemical biosensors^35,36^.

**Scheme 1:**
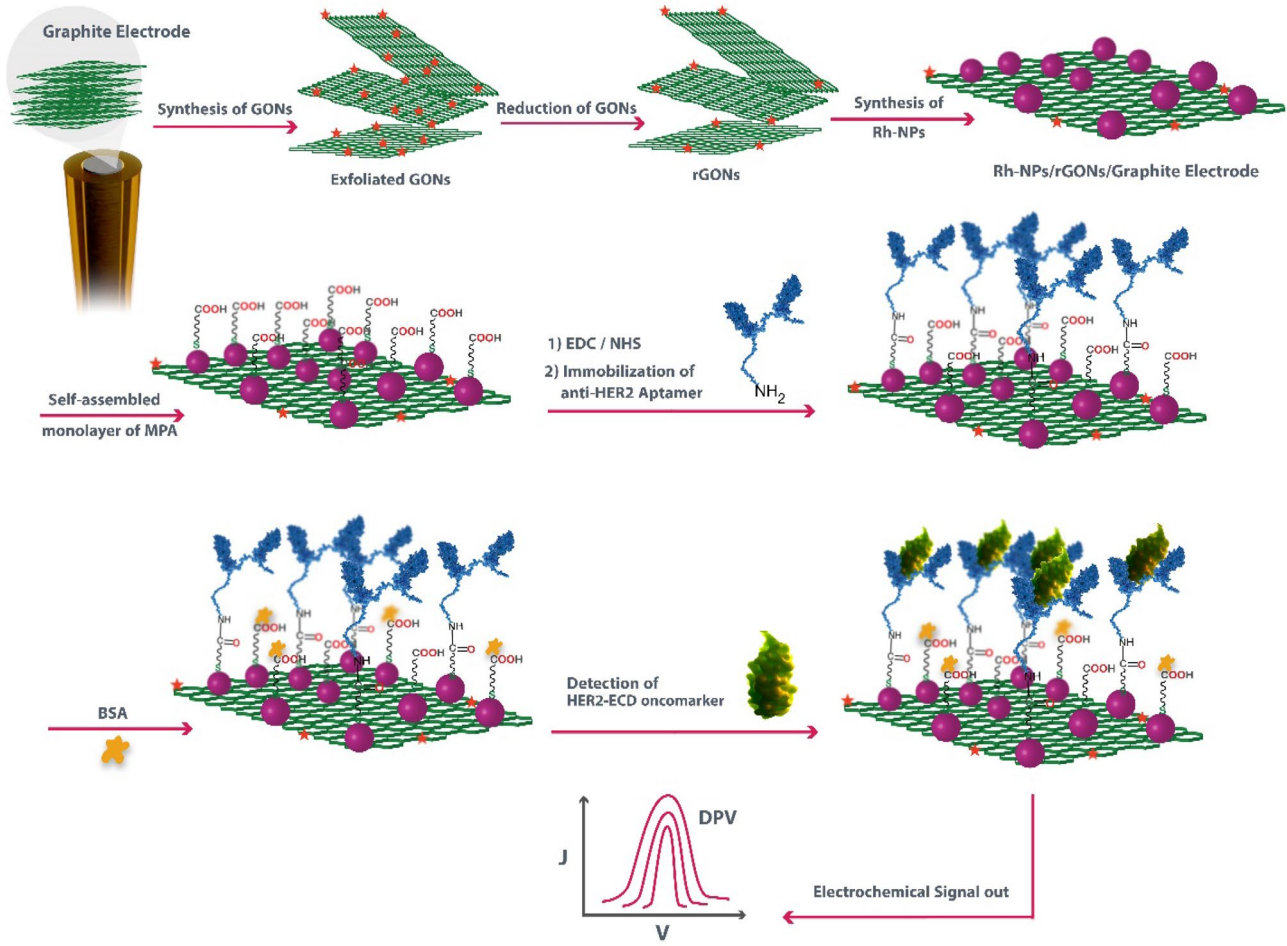
Schematic presentation of fabrication procedure of aptasensor for detecting HER2-ECD oncomarker: (1) the synthesis of rGONs, (2) the process of formation of Rh-NPs, (3) the immobilization of aptamer strands, and (4) the HER2-ECD detection using the proposed aptasensor.

In this study, we exploited an electrochemical aptasensing platform and employed a novel and efficient procedure to modify GE surface using rGONs and Rh-NPs functionalized with anti-HER2 aptamers for detection and quantification of HER2-ECD oncomarker. To the best of our knowledge, Rh-NPs have not been used for biosensor fabrication especially in diagnosis of cancerous oncomarkers (Scheme 1).

## Results and discussion

### Synthesis and preparation of the transducer nanomaterial

**Figure 1:**
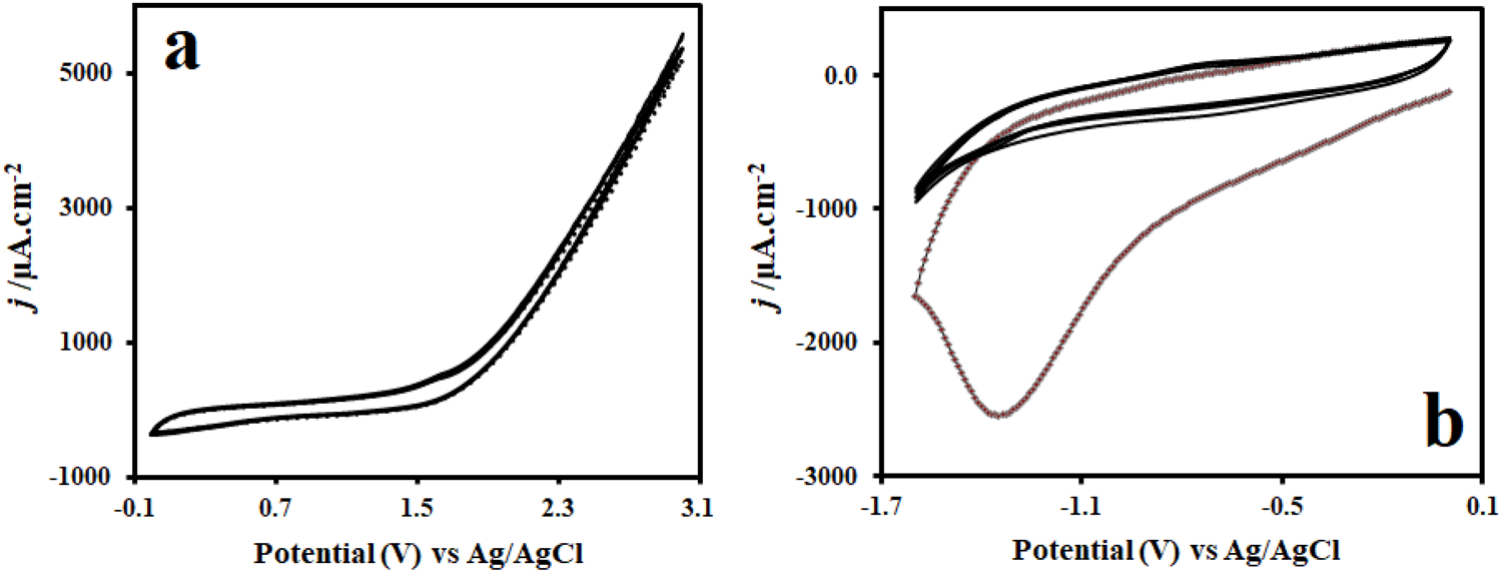
(**a**) CV curves of GONs synthesis on the GE for four consecutive sweeps in PBS (pH 6.5), (**b**) CV curves of rGONs synthesis on the GONs-modified GE for five consecutive sweeps in PBS (pH 6.9). Scan rate: 50 mV/s.

We synthesized rGONs on the GE by an eco-friendly in situ electrochemical method. To produce multi-layers of GONs on the GE surface, electrochemical oxidation and exfoliation were performed for four sequential sweeps in PBS (pH 6.5) at anodic potentials ranging from 0.0 to + 3.0 V. As shown in the cyclic voltammetry (CV) curves in Fig. 1a, electrochemical oxidation of graphite structure eventuated during the positive sweep at around + 1.4 V. During the electrochemical oxidation process, the van der waals and other cohesive forces in the midst of graphene sheets decreased and the layers separated and spaced apart with the help of gas evolution. Thus, intercalation and exfoliation of graphite to GONs occurred. Intercalating compounds comprising a diverse spectrum of hydrophilic oxygenated functional groups (i.e. hydroxyl, epoxy, carbonyl, carboxylic acid) were produced which the majority of them situated at the edges of basal planes^37,38^. CV scanning in cathodic potentials ranging from 0.0 to − 1.60 V for five sequential sweeps in PBS (pH 6.9) was used to electrochemically reduce the GONs to rGONs (Fig. 1b). The curve in the first cyclic voltammogram of electrochemical reduction shows a significant and broad cathodic peak current at about − 1.350 V. This significant peak is most likely due to reduction of oxygenated functional groups on the GONs-modified GE surface. This reduction can not be related to reduction of H_2_O molecules to hydrogen because it occurs at greater negative potentials. In the second CV, the reduction current peak decreases dramatically at negative potentials and almost disappears after the second scan. This finding suggests that a significant diminish in surface-oxygenated functional groups at GONs occurs and converts to rGONs irreversibly and rapidly. As a result, the exfoliated GONs can be reduced using electrochemical approach at negative potentials^39,40^. Accordingly, the number of voltammetric cycles, scan rate, pH, and temperature of the supporting electrolyte were among the characteristics effective in converting graphite to rGONs. This technique was more efficient when performed at middle scan rate (50 mV/s) and multiple voltammetric cycles to provide sufficient time for production of rGONs from graphite in two steps of electrochemical oxidation and reduction. At slightly acidic pH (6.5), the condition for oxidation process and formation of GONs were more favorable. However, it was better to have a slightly higher pH (6.9) during the reduction and conversion of GONs to rGONswhich reduces and eliminates the functional groups more easily and quickly. These experiments had the best results at ambient temperature (20 to 24 °C).

Noble metal nanoparticles (NMNPs) are efficiently produced by reducing the equivalent metallic salt in water^41,42^. Rh-NPs were synthesized using electrochemical reduction. In this procedure, Rh-NPs were grown on the surface under optimum conditions by applying CV technique in potentials between 0.0 and − 800.0 mV for four successive sweeps^43^. As a result, the rGONs-modified GE was adorned with Rh-NPs (Rh-NPs/rGONs/GE). For the synthesis of NMNPs, the rGONs provide numorous nucleating sites. Consequently, different procedures for the synthesis of graphene hybrids with noble metals such as Au, Ag, Rh, Pd, and Pt have been developed and investigated for design of electrochemical biosensors, catalysis, fuel cells, and so on^44^. The 2D structure of nano-sheets of graphene in these hybrid nanocomposites not only provides a perfect framework for anchoring the metal nanostructures, but also improve the electrical conductivity and interfacial electron transport liberated by these adherent NPs, limiting nanoparticle aggregation^45^.

### Morphology and spectroscopy characterizations

Field emission scanning electron microscopy (FE-SEM), Energy-dispersive X-ray spectroscopy (EDS), EDS Mapping, and Fourier transform infrared-attenuated total reflectance (FTIR-ATR) spectroscopy were used to analyze the as-prepared nanomaterials. All results indicated that the nanomaterials were successfully synthesized.

**Figure 2:**
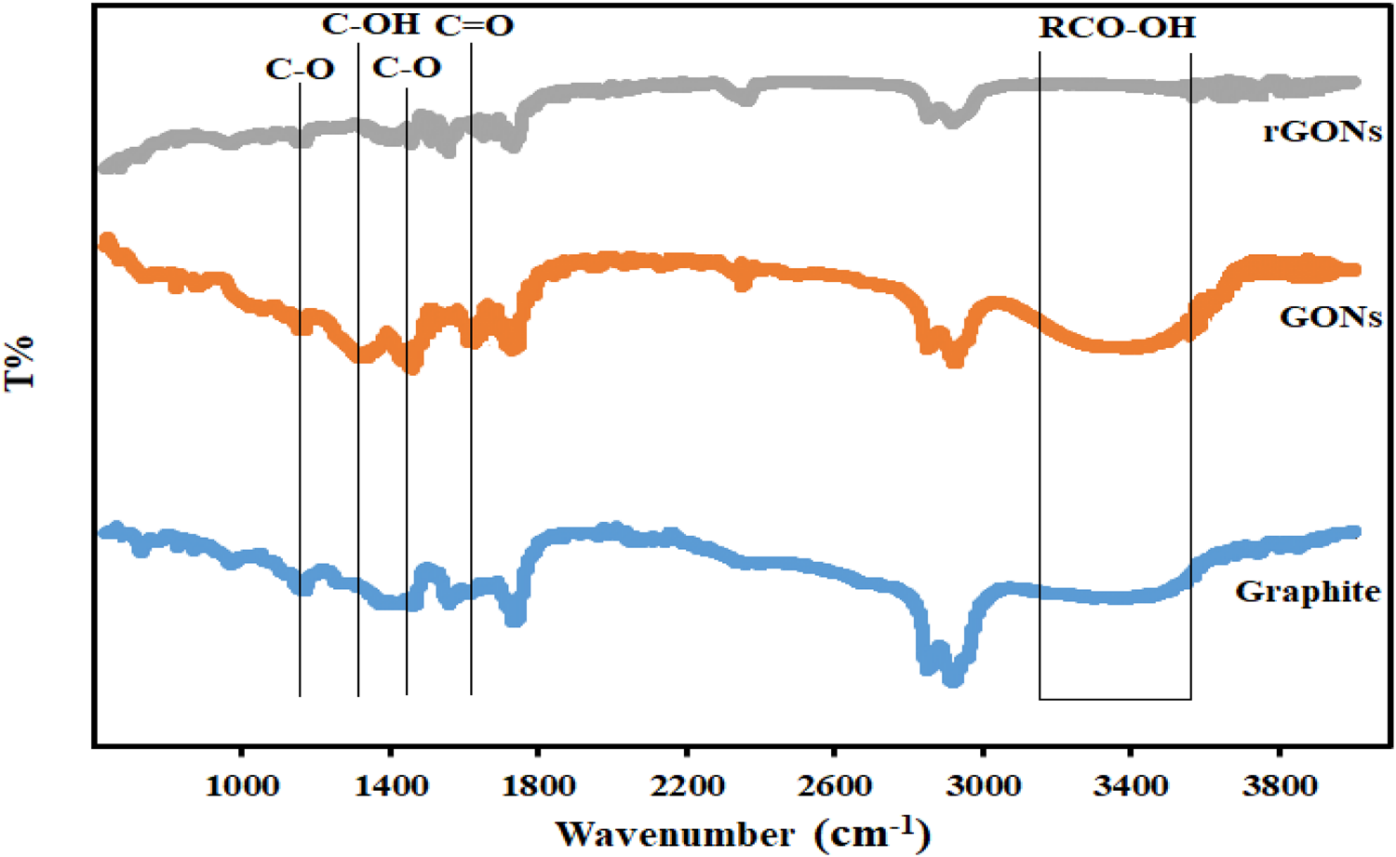
FTIR-ATR spectra of unmodified, GONs-modified, and rGONs-modified graphite electrodes (GEs).

The functional groups were identified using FTIR-ATR spectroscopic studies. The FTIR-ATR spectra of graphite, GONs, and rGONs are shown in Fig. 2. The characteristic peak at 1460 cm^−1^ corresponds to C=C skeletal vibration of graphitic domain. Several peaks in the GONs spectrum show oxygen-containing functional groups. The characteristic peaks of O–H (3391 cm^−1^), C=O (1732 cm^−1^), C–O (1556 cm^−1^), C–OH (1317 cm^−1^), and C–O (1162 cm^−1^) are thought to be assigned for stretching hydroxyl group in carboxylic acid, carboxyl group, carboxylic acid, and carbonyl group, respectively. The presence of oxygen functional groups in FTIR-ATR of GONs spectra indicates that the flake graphite has been oxidized to GONs. These peaks do not appear in the graphite spectra, showing the existence of a significant number of oxygen functional groups (–COOH and C=O near the sheet edge, –OH and C–O groups in epoxy on the GONs sheet basal planes) added during the oxidation step. There is no noticeable peak after reducing and converting GONs to rGONs, indicating that the rGONs are fully reduced. Carbon–oxygen functional groups such as carboxyl groupsremain in the rGONs structure with faint peaks.

**Figure 3:**
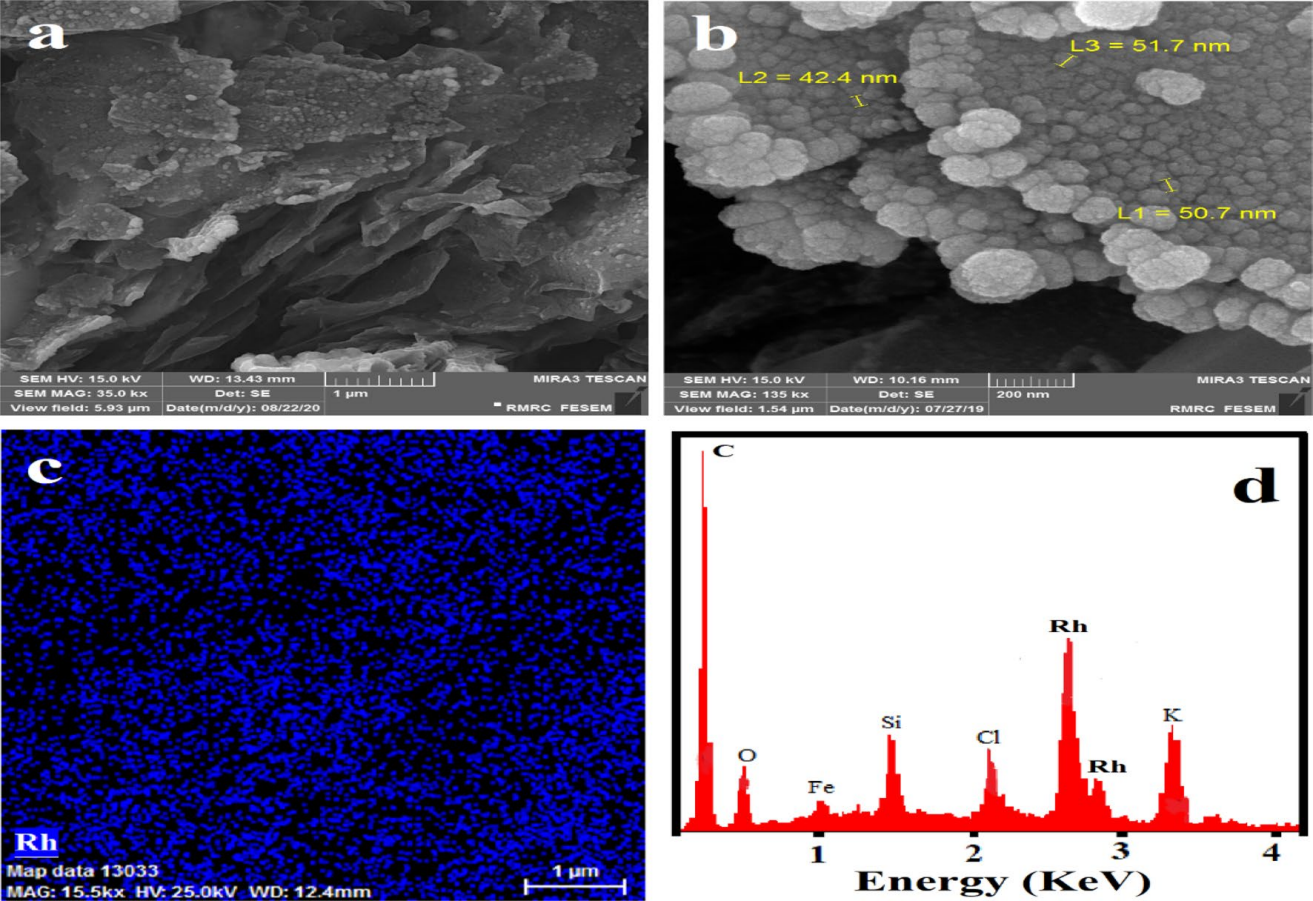
(**a**,**b**) FE-SEM images of Rh-NPs/rGONs/GE with nanometer-sized, (**c**) EDS mapping analysis of Rh element on the surface of rGONs-modified GE, (**d**) EDS elemental analysis obtained from Rh-NPs/rGONs/GE.

Figure 3a,b shows the FE-SEM images of Rh-NPs with spherical morphology extremely dispersed with nanoscale sizes that cover rGONs on the surface of graphite electrode. The elemental composition of the surface of Rh-NPs/rGONs/GE was also investigated by EDS mapping analysis, showingthat the modified electrode surface is coated with Rh-NPs (Fig. 3c). The EDS profile of carbon, oxygen, and rhodium (Rh) corroborates the successful formation of high-purity components (Fig. 3d).

### Electrochemical characterization of the synthesized nanomaterial

**Figure 4:**
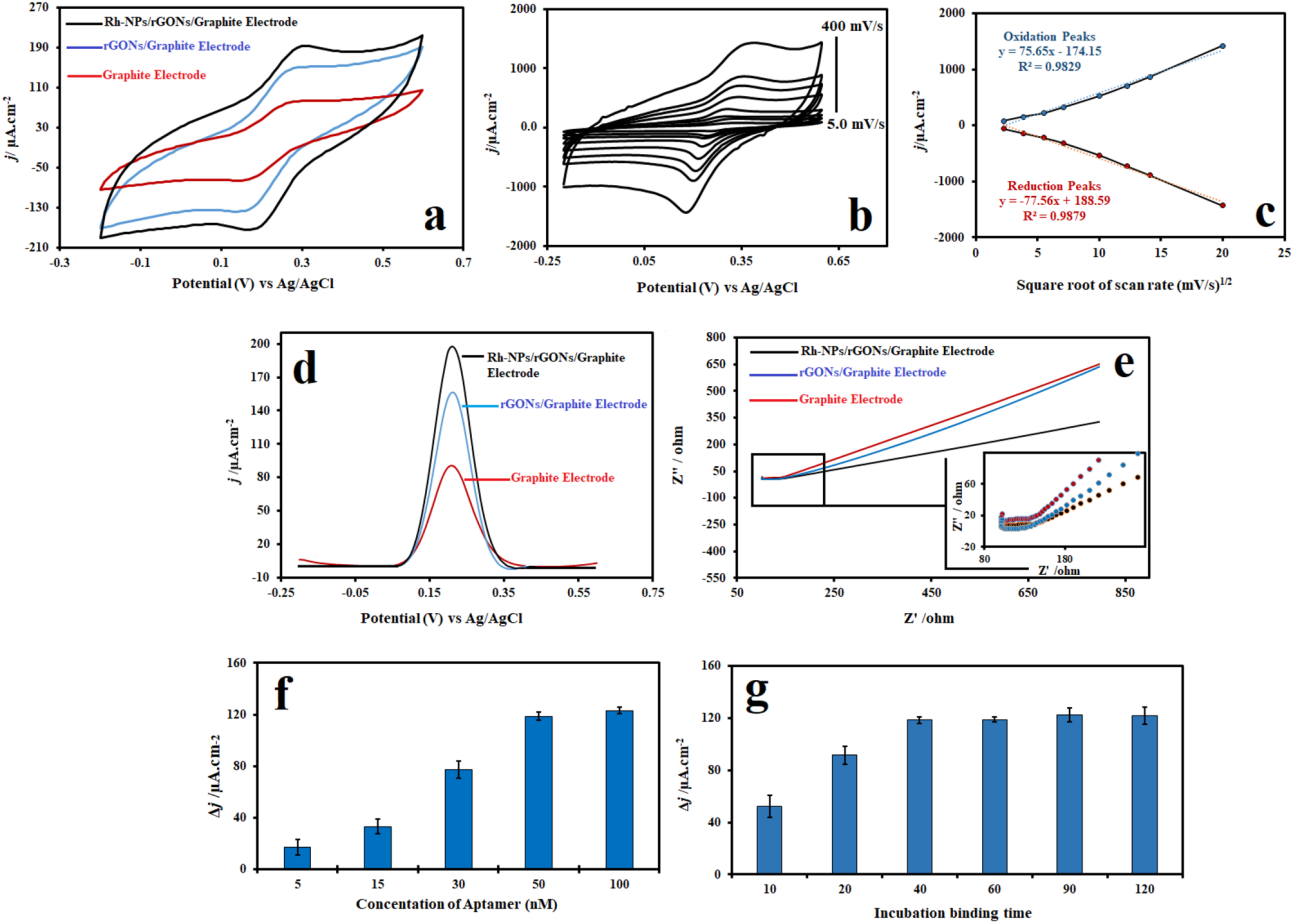
(**a**) Cyclic voltammograms of different unmodified and modifiedGE, (**b**) CV graphs of Rh-NPs/rGONs/GE based on various scan rates of 5, 15, 30, 50, 100, 150, 200, and 400 mVs^−1^, (**c**) Calibration curves of the redox peak current densities against the square root of the sweep rate based on cyclic voltammogram characterization of Rh-NPs/rGONs/GE, (**d**) DPV curves of different unmodified and modified GEs, (**e**) EIS Nyquist plots of different unmodified and modified GEs in PBS containing 5.0 mM ferri-/Ferro-cyanide (1:1) ion redox as an electrochemical probe, (**f**) Different concentrations of aptasensor against current densities, g) incubation binding time against current densities.

Electrochemical Impedance Spectroscopy (EIS), differential pulse voltammetry (DPV), and CV are essential techniques utilized in the development of biosensors and the evaluation of their performance^46^. The CVs of different modified GEs are shown in Fig. 4a. Electrochemical output of the modified GEs is compared with that of the unmodified GE. The CV technique is extensively implemented for exploratory purposes. The use of this technique in biosensor development is common since the CV technique possesses significant information such as types of redox processes present in the analysis and the process reversibility in reactions^47^. Redox peaks in the cyclic voltammogram correlated with the oxidation of ferrocyanide ion and reduction of ferricyanide ion on the GE surface are seen at 0.26 and 0.17 V, respectively. The peak current densities for the Rh-NPs/rGONs/GE are proportionately higher than those of the rGONs/graphite and bare GEs. This amplification in electrochemical signal could be attributed to increased conductivity in the presence of Rh-NPs and enhanced diffusive mass transport of the anion [Fe(CN)_6_]^4−^ to the Rh-NPs/rGONs/GEs surface compared with other unmodified and modified GEs. All electrochemical characterizations of the Rh-NPs/rGONs/GEs were carried out in PBS containing 5.0 mM Potassium ferri-/ferro-cyanide (+ 0.1 M KCl) as a solution of standard electrochemical probe.

Figure 4b demonstrates the peak currents in cyclic voltammogram for redox states in the presence of standard electrochemical probe at scan rates from 5.0 to 400.0 mV/s. As can be seen in Fig. 4c, there is a relationship of linearity between the square root of scan rate against the redox of peak current density. When the scan rate increases, the anodic and cathodic peak current densities elevate significantly at the same time with an average correlation coefficient (R^2^) value of 0.990, stating the redox reaction is diffusion controlled^48^.

The differential pulse voltammograms of unmodified and modified GEs are shown in Fig. 4d. After modifying the electrode with rGONs and Rh-NPs, peak current densities increase significantly. Electrochemical methods based on the pulse techniques like DPV are more sensitive than the linear sweep methods due to possible minimization of the interference capacitive current. The pulse techniques are mostly employed for quantitative determination since DPV has a significantly lower detection limit than other well-known electrochemical techniques owing to a greater signal-to-noise ratio.

EIS is a sensitive and precise electrical resistance measurement techniquegenerally employed for the characterization of modified electrode surfaces or significant change of bulk properties^49^. The electron transfer characteristics between electrolyte and electrode interfacial surface were checked using EIS in a solution of standard electrochemical probe. The charge transfer resistance (R_*ct*_) controls the kinetics of electron transfer of Potassium ferri-/ferro-cyanide ionic redox reaction at the electrolyte–electrode interface. When the GE is modified by rGONs and Rh-NPs (denoted as Rh-NPs/rGONs/GE), it exhibits a semicircle at the high frequencies including a small R_*ct*_ value that is the result of fast electron transfer^50^. Finally, among the different constructed GEs with various modifications, Rh-NPs/rGONs/GE was used as an optimal platform of aptasensor due to its high performance compared with others.

### Optimization and immobilization of anti-HER2 aptamer on the modified electrode

The predicted secondary structure of anti-HER2 aptameric strand shows that double stem-loops and a random sequence are in its primary stem-loop structure anticipated to be bound to the particular place of HER2-ECD. Transducer layer is extremely important for immobilization of the aptamer strand and stabilization of the formed G-quadruplex, affecting the sensitivity and selectivity of the aptasensor. It seems that nanomaterials are ideal platforms for immobilization of anti-HER2 aptamer strands due to their privileged bio-compatibility as well as extremely large surface area to volume ratio^51^. The interaction between HER2-ECD and aptameric strand leads to opening the anti-HER2 hairpin duplex and the formation of aptamer/HER2-ECD complex^52^. In this study a remarkable decrease in CV and DPV peak current measurements in aptasensor was seen. This is due to the fact thataptamers are insulating compounds that the phosphate groups in their structures would be ionized into plenty negative charges in aqueous solution that barricade the transfer of electron on the surface of electrode owing to the intense electrostatic repulsion with ferri-/ferro-cyanide ionic redox available in electrochemical probe^53^. The difference of electrochemical current density obtained by changing oncomarker concentration compared with BSA stabilization was usedas the measurement system displayed as Δ*j*. Then, the effect of two important parameters i.e., anti-HER2 aptamer concentration and incubation binding time for HER2-ECD oncomarker was explored. First, the MPA/Rh-NPs/rGONs/GEs which have already been activated by EDC/NHS were submerged in different concentrations of anti-HER2 aptamer (5, 15, 30, 50, and 100 nM), dipped in 5% BSA solution, and incubated with 300 ng/mL of HER2-ECD. As clearly exhibited in Fig. 4f, an increase in the concentration of anti-HER2 aptamer up to 50 nM enhances the current density. However, no significant change is observed for aptamer concentration greater than 50 nM since the modified surface of electrode is thoroughly saturated with anti-HER2 aptamer^54^. Accordingly, a concentration of 50 nM anti-HER2 aptamer was selected as the optimal concentration. Second, to gain the best incubation binding time of the HER2-ECD, the aptasensor in concentration of 300 ng/mL of was incubated for 10, 20, 40, 60, 90, and 120 min. The intensity of current density (*j*) raised with increasing the incubation binding time and then stayed almost steady after 40 min incubation because the surface of electrode was saturated. For this reason, the most favorable incubation time was determined to be 40 min (Fig. 4g).

### HER2-ECD biosensing performance of aptasensor

Since the selected anti-HER2 aptamer strand was made up of 54 oligonucleotide bases (5′-(NH_2_-(CH_2_)_6_-GGG CCG TCG AAC ACG AGC ATG GTG CGT GGA CCT AGG ATG ACC TGA GTA CTG TCC)-3′)^51,55^, optimizing the sensitive layer to bind the aptamer strand to surface of modified GE and immobilize the generated G-quadruplex was critical. Given their high bio-affinity and intrinsic bio-compatibility, rGONs and Rh-NPs could be used as a platform for immobilizing anti-HER2 aptamer strands, followed by HER2-ECD recognition via G-quadruplex formation between aptamer strands and HER2-ECD oncomarker. As a consequence, the aptasensor based on BSA/Apt/MPA/Rh-NPs/rGONs/GE was employed to evaluate each stage of the detection procedure of various oncomarkers.

### Biosensing performance of aptasensor toward HER2-ECD oncomarker

**Figure 5:**
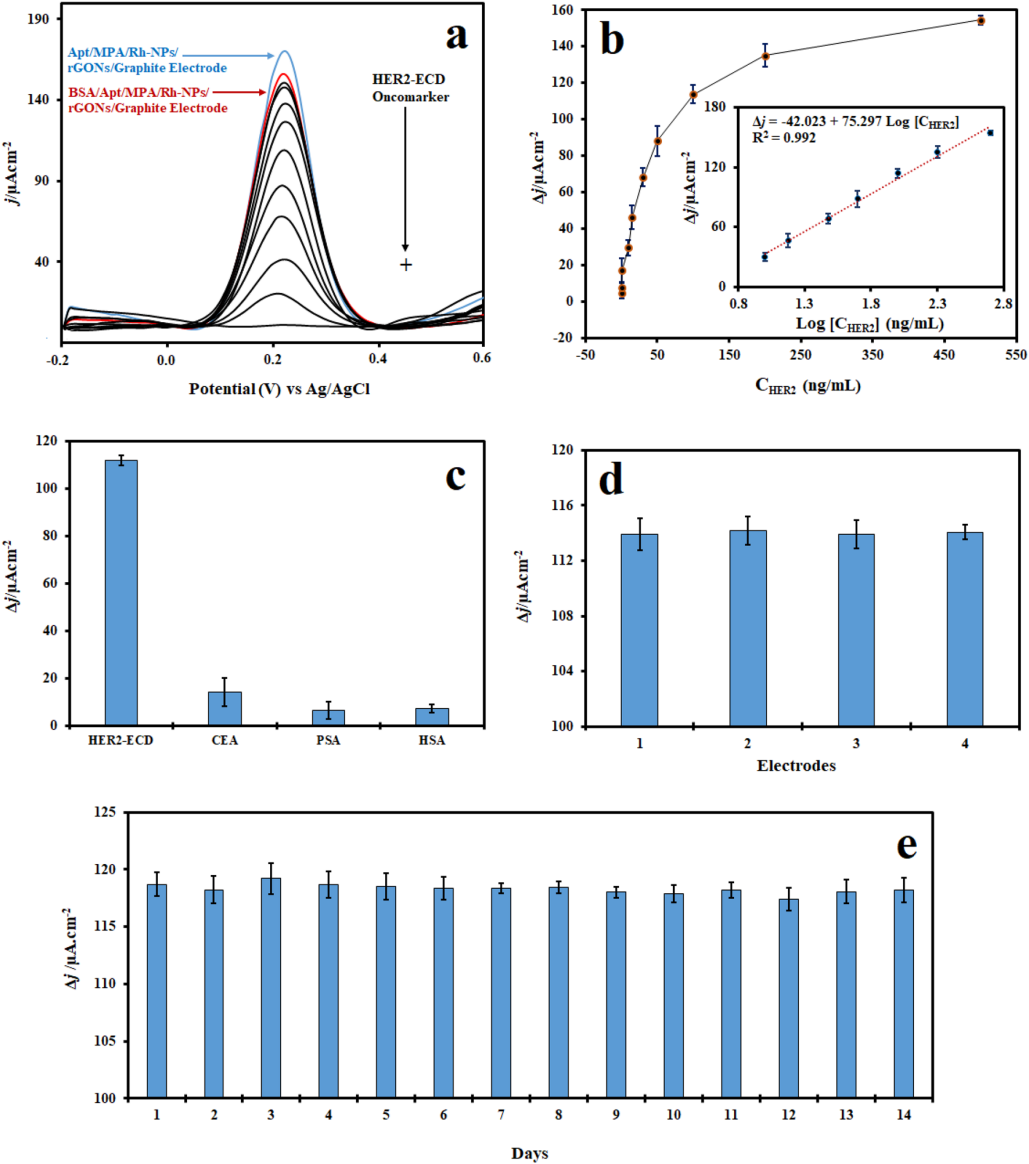
(**a**) DPV responses of aptasensor with different concentrations of HER2-ECD (0.01, 0.1, 1, 10, 15, 30, 50, 100, 200, 500 ng/mL); (**b**) Dependence of current density on changes in HER2-ECD concentration (inset: the linear portion of calibration curve); (**c**) Selectivity of aptasensor in contact with 100 ng/mL HER2-ECD, CEA, PSA, and HSA oncomarkers; (**d**) Reproducibility of aptasensor for detection of 100 ng/mL HER2-ECD with 4 electrodes, separately; (**e**) Stability of aptasensor for detection of 300 ng/mL HER2-ECD within 14 days, Each measurement was repeated at least 4 times.

The proposed aptasensor was also employed to detect HER2-ECD oncomarker at various doses. For this purpose, HER2-ECD oncomarker was tested at concentrations ranging from 0.010 to 500.0 ng/mL. Then, the interaction results were measured using the DPV technique (Fig. 5a). As demonstrated in Fig. 5b, by rising the concentration of HER2-ECD, the current density increases. The electrochemical response of the aptasensor towards HER2-ECD is in the linear range of 10.0 to 500.0 ng/mL. The linear relationship between Δ*j* and Log [C_HER2-ECD_] was: Δ*j* (µA cm^−2^) = − 42.023 + 75.297 Log [C_HER2-ECD_] (ng/mL) and an R^2^ of 0.9937, where [C_HER2-ECD_] is the concentration of HER2-ECD. The LOD and LOQ calculated using this equation is 0.665 ng/mL and 2.01 ng/mL, respectively. This aptasensor provides a satisfactory performance compared with the present HER2-ECD biosensors described in the literature (Table 1). The significant stability of the aptasensor can be ascribed to the BSA/MPA/Rh-NPs/rGONs/GE and strong covalent binding of anti-HER2 aptamer to the modified electrode via MPA linker. Therefore, the results demonstrates good performance of aptasensor even at lower HER2-ECD oncomarker concentrations that can be employed to monitor small changes in oncomarker concentration in many forms of cancer during the primary stage of disease. The selectivity of electrochemical aptasensor was also investigated using different interfering agents such as carcinoembryonic antigen (CEA), prostate-specific antigen (PSA), and Human Serum Albumin (HSA). The results show that PSA and CEA have a slight change in current density (Fig. 5c) whereas the HER2-ECD oncomarker has a significant change. Four electrodes were made to evaluate reproducibility (Fig. 5d). These findings show that the aptasensor is exceedingly selective and stable for direct detection of the HER2-ECD oncomarker. The long-term stability is an important parameter in clinical applications. As shown in Fig. 5e, the signal changes after 14 days are minimal and this indicates that the aptasensor has maintained its detection performance and stability compared with the first day with relative standard deviation (RSD) 4.3%. Therefore, the developed aptasensor has a high level of reliability and efficiency.

**Table 1:** Comparison of the suggested electrochemical aptasensor to other constructed biosensors towards detection of HER2-ECD oncomarker.

Materials	Technique	Linear range (ng/mL)	LOD (ng/mL)	References
Screen-printed carbon electrode (SPCE)/Ab/BSA/HER2-ECD/ /labeled Ab with CdSe@ZnS Quantum Dots (QDs)	DPV	10–150	2.1	^56^
Au electrode (AuE)/Tetrahedral DNA nanostructures-aptamerHER2-ECD/Mn_3_O_4_.pd@pt.horseradish peroxidase (HRP) (nanoprobe 2)/pd@pt.HRP.complimentary DNA (nanoprobe 1)	DPV	0.1–100	0.08	^57^
SPCE/Biomodified carboxylic acid functionalized magnetic beads (COOH-MBs)/Ab/BSA/HER2-ECD/Biotinylated Ab/Alkaline phosphate/Silver ions	LSV	5.0–50 and 50–100	2.8	^58^
/Glassy carbon electrode (GCE)/Functionalization of 3-aminopropyltrimethoxysilane (APTMS) coated Fe_3_O_4_ NPs with Ab/BSA/HER2-ECD/Ab.Hydrazine@AuNPs.APTMS. Fe_3_O_4_**/**Reduced silver ions	DPV	5.0 × 10^−4^–50.0	2.0 × 10^−5^	^59^
Interdigitated AuE/Thiol terminated DNA aptamer/Saline-Tween 20/HER2-ECD	EIS	0.1–10^4^	0.1	^19^
AuE/Peptide specific to HER2-ECD/HER2-ECD/immobilized Ab and Polycytosine DNA sequence on the AuNPs/molybdate	Square wave voltammetry (SWV)	0.001–1	0.0005	^60^
AuE/Bimetallic ZrHf metal–organic framework/Aptamer/BSA/HER2-ECD	EIS DPV	0.0001–10	0.000030 0.000019	^51^
GCE/Fe_3_O_4_@TMU-21/Multi-walled carbon nanotube/Ab/BSA/HER2-ECD	Amperometry	0.001–100	0.0003	^61^
GCE/Cobalt porphyrin binuclear framework/Gold functionalized graphene QDs/Ab/BSA/HER2-ECD	EIS	0.1–10	0.0327	^62^
GE/rGONs/Rh-NPs/aptamer/HER2-ECD	DPV	10–500	0.665	This work

### Real sample analysis

The practical applicability of the introduced aptasensor was examined and approved by the electrochemical analyses of oncomarkers present in patient serum samples. The aptasensor was employed for detection and quantification of HER2-ECD in serum samples from five patients. The results (Table 2) were in agreement with commercially available ELISA kit. Hence, it can be deduced that the proposed aptasensor is reliable and sensitive enough for clinical application.

**Table 2:** HER2-ECD oncomarker analysis in human serum samples.

Serum sample no	Concentration (ng/mL)		RSD (%)	*P* value
	Proposed aptasensor	ELISA kit		
1	56.3 ± 0.26	59.8 ± 0.25	0.92	< 0.0001
2	28.6 ± 0.34	23.4 ± 0.23	2.4	< 0.0001
3	43.5 ± 0.36	42.7 ± 0.29	1.2	0.63
4	79.8 ± 0.27	81.4 ± 0.20	0.69	0.049 (< 0.05)
5	89.5 ± 0.64	88.8 ± 0.81	0.88	0.69

The results are the average of four independent measurements ± standard error.

## Conclusion

In summary, a novel and well-organized aptasensor was constructed for detection and determination of HER2-ECD oncomarker. The collaborative effect of rGONs and Rh-NPs can boost electrochemical signal and sensitivity by increasing conductivity and specific surface area. Furtheremore, the stability of the formed G-quadruplex increased through strong covalent interactions between the aptameric strand and the oncomarker. Accordingly, a low value of LOD was obtained equal to 0.667 ng/mL. The results of this study confirmed the successful performance of the constructed aptasensor. This promising electrochemical aptasensor could be feasibly applied as a platform for diagnosis and monitoring of a wide variety of oncomarkers in different cancers.

## Experimental methods

### Materials and chemicals

The pencil graphite type HB with a diameter of 2.0 mm was purchased from Rotring Co. Ltd, Germany. 3-Mercaptopropionic acid (MPA), N-HydroxySuccinimide (NHS), Bovine Serum Albumin (BSA), Human Serum Albumin (HSA) 1-ethyl-3-(3-dimethylaminopropyl) Carbodiimide hydrochloride (EDC), Rhodium(III) Chloride (RhCl_3_, 98%), and HER2 ELISA assay Kit (for serum, plasma, urine, and etc.) was procured from Sigma-Aldrich, USA. Potassium ferricyanide (k_3_Fe(CN)_6_), and Potassium ferrocyanide (K_4_Fe(CN)_6_) were obtained from Merck, Germany. Anti-HER2 aptamer specifically bind to HER2-ECD oncomarker was obtained from Bio Basic Inc, Canada. The sequence of Amine-terminated HER2 DNA aptamer (Apt) consists of 54-mer oligonucleotide bases is 5′-(NH_2_-(CH_2_)_6_-GGG CCG TCG AAC ACG AGC ATG GTG CGT GGA CCT AGG ATG ACC TGA GTA CTG TCC)-3′. This anti-HER2 DNA aptamer sequence was selected by serial evolution of ligands in vitro by exponential enrichment (SELEX) process^51,55^. The recombinant human HER2/ErBb2/CD340 oncomarker was procured from Sigma-Aldrich, USA. Sulfuric Acid (H_2_SO_4_ 98%), Hydrochloric Acid (HCl 37%), and Ethanol (99.8%), were purchased from Merck, Germany. Other analytical grade reagents were obtained of the highest level of purity and all required solutions were prepared from double distilled water.

### Electrochemical measuring equipment and instruments

All electrochemical measurement include CV, DPV, and EIS were carried out using a potentiostat–galvanostat Palmsense BV PGSTAT, with a compliance voltage of 30 V (Echo chemie, Netherlands) by a customary three-electrode cell arrangement. The graphite with various modifications was employed as a working electrode. The desired amplitude of potentials were measured against an Ag/AgCl in a solution containing saturated KCl, acting as the reference electrode. A platinum wire electrode was used, functioning as counter electrode. A freshly polished and prepared electrode was used for each experiment. The measurements were performed exactly after applying the three electrodes and immersing them in the probe solution. All electrochemical experiments were carried out in PBS (0.1 M, pH 7.4) containing of 5.0 mM K_4_[Fe(CN)_6_]/K_3_[Fe(CN)_6_] (1:1 ratio) and 0.1 M KCl as ion redox probe couple. The ferri-/ferro-cyanide ionic redox pair in buffered media is frequently employed as a standard probe in electrochemical studies. The [Fe(CN)_6_]^3−/4−^ ionic redox process involves a single electron transfer and exhibits quasi-reversible kinetic characteristics under the condition that the electron transfer in a complete cycle is less resistant^63^.

CV curves were recorded with a scan rate of 50 mV/s and within − 0.2 to + 0.6 V vs Ag/AgCl. DPV was measured between − 0.2 to + 0.6 V, with an amplitude of 50 mV, a pulse width of 0.2 s. EIS measurements were carried out in the frequency range of 0.01 to 50 kHz with a direct potential of 0.22 V as a bias potential and an amplitude of 5.0 mV.

### Synthesis of reduced graphene oxide nano-sheets

We used a simple, efficient, and novel in situ redox electrochemical approach to directly produce rGONs-modified GEs. Briefly, Cyclic Voltammetry (CV) sweeping the potential from 0.0 to + 3.0 V was achieved for a series of four successive sweeps in PBS (pH 6.5) to form few-layer GONs on the surface of electrode. Later, CV sweeps carried out from 0.0 to − 1.60 V in PBS (pH 6.9) for electrochemical reduction of exfoliated GONs in five successive cycles to obtain rGONs-GE^39,64,65^.

### Synthesis of rhodium nanoparticles on the surface ofGE

Rh-NPs were electrochemically synthesized using an electrolyte solution containing 0.01 M RhCl_3_ and 0.01 M KCl. CV technique was employed in the potential range from 0.0 to − 800.0 mV for four successive sweeps. The electrolyte solution was provided in double-distilled water to a final volume of 10 ml. The cathodic reduction of rhodium ions at room temperature occurs as follows:

$${\text{Rh}}_{{{\text{aq}}}}^{{{\text{3 + }}}} {\text{ (RhCl}}_{{\text{3}}} {\text{) + 3e}}^{ - } \to {\text{Rh}}^{0} {\text{.}}$$

### Preparation of the GE-based aptasensor

In order to prepare the electrode used in aptasensor, the Rh-NPs/rGONs modified GE was suspended in 20 mM MPA solution dissolved in ethanol/water (3:1 V/V) at pH 6.8 under shaking (75 rpm) at room temperature for 18 h. Thus, MPA/Rh-NPs/rGONs/GE was rinsed with double-distilled water. The optimal concentration of the MPA was used as a linker to bind modified electrode to the bio-recognition agent (anti-HER2 aptamer). The carboxyl-functionalized MPA can strengthen the binding of amino-functionalized strands of aptamer as well as enhance electrochemical activity of the aptasensor^66^. The MPA/Rh-NPs/rGONs/GE was incubated in PBS containing 0.05 M NHS and 0.20 M EDC for 1 h at 8 °C in order to activate its surface. For covalent conjugation of activated surface with amine group (-NH2) of functionalized anti-HER2 aptamer, the modified electrode was dipped in PBS buffer containing 50 nM anti-HER2 aptamer at room temperature for 1 h, for amide bond formation. Furthermore, Apt/MPA/Rh-NPs/rGONs/GE was immersed in BSA solution with concentration of 5% at the temperature of 37 ºC under 5% CO_2_ atmosphere and humidity with 95% for 5 min to block the activated carboxyl functional groups on the surface of modified electrode that are not bonded with the amino functional groups of anti-HER2 aptamer. The functionalized GE was rinsed with PBS for several times. Finally, the constructed aptasensor was employed to detect different oncomarkers.

### Preparation of solutions

8.0 g NaCl, 200 mg KCl, 1.44 g Na_2_HPO4 and 245 mg KH_2_PO4 were combined to make a 1.0 L phosphate buffered solution (PBS, 0.1 M, pH 7.4). In the aforesaid PBS, a stock solution of aptamer (100 M), HER2-ECD, CEA, PSA, and HSA was produced and kept at 4 °C. In addition, the solution was diluted with PBS to achieve the appropriate concentration. For real-time analysis, the human serum was diluted 20-fold with 0.01 M PBS solution (pH 7.4). Also, for the synthesis of rGONs, the pH of the 0.1 M PBS was adjusted by 0.01 M HCl.
